# The Procedural Index for Mortality Risk (PIMR): an index calculated using administrative data to quantify the independent influence of procedures on risk of hospital death

**DOI:** 10.1186/1472-6963-11-258

**Published:** 2011-10-07

**Authors:** Carl van Walraven, Jenna Wong, Carol Bennett, Alan J Forster

**Affiliations:** 1 Clinical Epidemiology Program, Ottawa Hospital Research Institute, 1053 Carling Avenue, Ottawa, K1Y 4E9, Canada; 2 Institute for Clinical Evaluative Sciences, 1053 Carling Avenue, Ottawa, K1Y 4E9, Canada; 3 Department of Medicine, University of Ottawa, 1053 Carling Avenue, Ottawa, K1Y 4E9, Canada

## Abstract

**Background:**

Surgeries and other procedures can influence the risk of death in hospital. All published scales that predict post-operative death risk require clinical data and cannot be measured using administrative data alone. This study derived and internally validated an index that can be calculated using administrative data to quantify the independent risk of hospital death after a procedure.

**Methods:**

For all patients admitted to a single academic centre between 2004 and 2009, we estimated the risk of all-cause death using the Kaiser Permanente Inpatient Risk Adjustment Methodology (KP-IRAM). We determined whether each patient underwent one of 503 commonly performed therapeutic procedures using Canadian Classification of Interventions codes and whether each procedure was emergent or elective. Multivariate logistic regression modeling was used to measure the association of each procedure-urgency combination with death in hospital independent of the KP-IRAM risk of death. The final model was modified into a scoring system to quantify the independent influence each procedure had on the risk of death in hospital.

**Results:**

275 460 hospitalizations were included (137,730 derivation, 137,730 validation). In the derivation group, the median expected risk of death was 0.1% (IQR 0.01%-1.4%) with 4013 (2.9%) dying during the hospitalization. 56 distinct procedure-urgency combinations entered our final model resulting in a Procedural Index for Mortality Rating (PIMR) score values ranging from -7 to +11. In the validation group, the PIMR score significantly predicted the risk of death by itself (c-statistic 67.3%, 95% CI 66.6-68.0%) and when added to the KP-IRAM model (c-index improved significantly from 0.929 to 0.938).

**Conclusions:**

We derived and internally validated an index that uses administrative data to quantify the independent association of a broad range of therapeutic procedures with risk of death in hospital. This scale will improve risk adjustment when administrative data are used for analyses.

## Background

Surgeries and procedures are major functions of hospitals that importantly influence patient outcomes and hospital performance. Procedural outcomes are often used to compare surgeons, clinical divisions, hospitals, and health jurisdictions. Many different types of surgeries and procedures exist in different specialties, involving very different patient populations. As a result, the influence of different types of procedures on hospital outcomes can vary greatly.

Quantifying the independent influence of a broad range of different types of procedures on outcomes would allow analysts, administrators, and researchers to measure, compare, and adjust for the importance of each procedure. Six indexes have been developed to quantify the risk of post-operative death after a range of surgeries (Table 1) [1-6]. Each of these indexes, however, requires clinical information that is usually unavailable in routinely collected administrative data.

**Table 1:** Summary of previous indexes predicting risk of death following surgery

Index	Study Population (N derivation/N validation)	Variables in model	Validation Area Under the Curve
P-POSSUM[6]	General surgical patients (2500/7500)	Age, cardiac history, respiratory history, blood pressure, pulse rate, Glascow Coma Score, labs (hemoglobin, WBC, urea, Na+, K+, ECG); operative severity, mulitple procedures, total blood loss, peritoneal soiling, presence of malignancy, mode of surgery	-

SRS[5]	Patients of three general surgeons (3144/2780)	CEPOD classification; BUPA operative grade; ASA score	0.95 (0.93, 0.97)[5]

Cr-POSSUM[1]	Patients having emergency or elective colorectal surgery (4079/2691)	Age; cardiac failure; SBP; pulse; urea; haemoglobin; operative severity; peritoneal soiling; operative urgency; cancer staging	0.90 (0.88, 0.92)[1]

Nottingham[3]	65+ years, acute or elective surgical and acute urological patients undergoing operative or conservative treatment (2923/1362)	Age; white cell count; urea; pulse rate; mean systolic and diastolic blood pressure; emergency admission; emergency operation; major operation; vascular disease; malignant disease; conservative treatment	0.86 (0.82, 0.89)[3]

AFC[25,26]	Undergoing open or laparoscopic surgery for colorectal cancers or diverticular disease (1421/395)	Emergency surgery (surgery withing 24 hours of admission); weight loss > 10% within past 6 months; neurological disease history; age > 70 years	0.89[4]

E-POSSUM[2]	65+ years; undergoing first colorectal operation or early colorectal reoperation (791/395)	Age group (WHO classification); physiological score (cardiac history, respiratory history, blood pressure, pulse rate, Glascow Coma Score, haemoglobin level, white cell count, urea concentration, Na+ level, K+ level, electrocardiography); operative severity score (operative severity, mulitple procedures, total blood loss, peritoneal soiling, presence of malignancy, mode of surgery)	0.86 (0.81, 0.92)[2]

CEPOD - Confidential Enquiry into Perioperative Death

BUPA - British United Provident Association

ASA - American Society of Anesthesiologists

In this study, we derived and internally validated an index to measure the influence of a broad range of surgeries on in-hospital mortality. Our goal was to quantify the independent association of all procedures with the risk of death in hospital. To do this, we first grouped procedures based on administrative codes and the procedure's urgency status and then determined which of these procedure-urgency groups were associated with risk of death in hospital after adjusting for factors that are highly predictive of this outcome. We then created a scoring system to quantify the independent association of significant procedures with risk of death in hospital. This index can be calculated using administrative data and estimates the risk of death in hospital from these procedures that is independent of other factors associated with this outcome. It can be used to help risk-adjust analyses using administrative data that have death in hospital as an outcome. Such analyses could be done to identify factors independently associated with death in hospital and, in some situations, compare quality of care between institutions.

## Methods

### Study Setting

This study took place at The Ottawa Hospital (TOH), a tertiary-care teaching facility with three sites that averaged 20 000 admissions annually during the study period. TOH functions within a publicly funded health care system. TOH is the sole regional provider of trauma care, thoracic surgery, and neurosurgical interventions, and provides most of the region's oncological care.

### Patients

We included all admissions to the hospital (including same-day surgeries) between 1 April 2004 and 1 April 2009. "Same-day surgeries" included patients who had their surgery on the same day on which they were admitted to hospital. These patients were typically discharged home the same day but may have been kept in hospital if complications occurred or if additional monitoring was required. We started patient recruitment in April 2004 to ensure that our hospital had at least two years of experience coding procedures with the Canadian Classification of Interventions (CCI) coding system (which was introduced in April 2002). Patient recruitment ended in April 2009 (the last complete year of data available when the analyses were conducted). To apply the Kaiser Permanente In-patient Risk Adjustment Model (KP-IRAM) [7] - the method used to adjust for other risk factors associated with death in hospital - we excluded all patients with age ≤ 15 years at admission, all delivery-related obstetrical admissions, and those who were transferred to or from TOH. Throughout this study, the unit of analysis was the hospitalization.

### Candidate Procedures

We used multiple binomial logistic regression to derive our index. We chose death in hospital as the model outcome because it is accurately recorded and is important to all potential users of the index. There were a total of 4013 hospital deaths (2.9% of all admissions) in the derivation cohort. Our logistic model could therefore test a maximum of 400 procedures or surgeries (i.e. 10 deaths per exposure) to safely avoid problems with over-fitting and model instability [8].

We identified candidate procedures using their Canadian Classification of Interventions (CCI) code. The CCI system contains more than 18,000 unique codes. We therefore grouped procedures using the first five alpha-numerics of each code (which identifies the anatomical area and the intervention type) and limited our study to therapeutic procedures (i.e. CCI section 1). We used the admission status of the hospitalization (i.e. elective vs. non-elective admission) to classify the procedure urgency since urgency is an important and independent predictor of post-procedural outcomes [9-14]. Procedures that could not be performed electively (such as cardiac resuscitation, implantation of an internal device in the thoracic descending aorta, and control of bleeding in the thoracic cavity) were classified as "non-elective" regardless of the admission status of the hospitalization.

There were 3984 unique procedure-urgency combinations during the study period. Since this exceeded the maximum number of variables allowed in our model without overfitting (n = 400), we used three filters to exclude procedures. First, we only included procedures that were conducted on the day of the principal procedure (defined as the procedure considered by the health records analyst to be most significant during the patient's hospital stay). In 5% of hospitalizations, coded procedures occurred on more than one day. In such cases, only procedures that occurred on the day of the principal procedure were considered. Second, procedures had to be conducted at least once per month at our hospital during the study period (independent of its urgency status). Finally, the p-value for the association of the procedure with death in hospital (after adjusting for risk of death in-hospital measured with KP-IRAM) had to be less than 0.5.

### Adjusting for Risk of Death in Hospital

To adjust for risk of death in hospital due to patient and hospitalization factors, we used the Kaiser Permanente In-patient Risk Adjustment Model (KP-IRAM) [7]. This model was derived and internally validated on almost 260,000 hospitalizations at 17 hospitals belonging to the Kaiser Permanente Health Plan and was subsequently validated at our hospital [15]. The KP-IRAM includes six covariates including: patient age; patient sex; admission urgency (i.e. elective or emergent) and service (i.e. medical or surgical); admission diagnosis; severity of acute illness as measured by the Laboratory-based Acute Physiology Score (LAPS); and chronic comorbidities measured by the Comorbidity Point Score (COPS). Using the admission diagnosis, hospitalizations were grouped into "Primary Conditions," and a separate logistic regression model was created for each group. Interaction terms between age, LAPS, and comorbidity score were included. The model had excellent discrimination (c-statistic = 0.88) and calibration (p-value of Hosmer Lemeshow statistic for the entire cohort was 0.66) for all-cause death in hospital.

We made three minor modifications to the KP-IRAM for this study. First, Canada switched from the International Classification of Diseases (ICD) 9-CM system (used in the KP-IRAM) to the ICD-10-CA system in 2002. We therefore used tables (provided by Canadian Institute for Health Information) to translate ICD-9-CM admission diagnoses to ICD-10-CA codes. Second, we measured chronic comorbidities using the Elixhauser Index [16] instead of the COPS because the KP-IRAM performed equally well using either comorbidity index [15]. Finally, the KP-IRAM was calculated on the day of the procedure (rather than at admission) for people who had one of the procedures included in the model. This model was used to estimate each patient's risk of death in hospital at the time of the procedure (expressed as a number that ranged between 0 and 1).

### Creation of the Procedural Index for Mortality Risk (PIMR) Score

We randomly separated patients into equally sized derivation and validation groups. Using the derivation group, we ran a binary logistic regression model with death in hospital as the outcome and the KP-IRAM estimated risk as the adjusting covariate. The index day for patients undergoing one of the procedures considered for the model was the day of the procedure. For all other patients, the index day was the day of admission. Values of all covariates for the KP-IRAM model were those on the index day. We used stepwise variable selection to identify which candidate procedure-urgency combinations were independently associated with death in hospital. Surgeries with a 2-sided p-value less than 0.05 were retained in the model.

We then used the methods described by Sullivan et. al. [17] to modify the parameter estimates of this regression model into an index. The number of points assigned to each procedure equaled its regression coefficient divided by the coefficient in the model with the smallest absolute value. We rounded this quotient to the nearest whole number. This number translated the parameter estimates into units relative to the procedure with the smallest, independently significant association with death in hospital. Therefore, the association of a procedure assigned two points was twice as important for predicting risk of death in hospital as a procedure with one point. Each person's total Procedural Independent Mortality Risk (PIMR) score was then calculated by summing up the points of all significant procedural groups for which they had been coded.

When calculating the PIMR score, we tallied up only those procedures that were performed on the index day (i.e. the day on which the principal procedure was conducted). Procedures done on other days did not influence the PIMR score. The PIMR score also did not capture whether or not the procedure was the first procedure conducted during the hospitalization.

### Assessment of the PIMR score

In the validation group, we described the distribution of the PIMR score and used logistic regression to measure the association of the PIMR score alone with risk of death in hospital.

We then measured the influence of the PIMR score on risk of death in hospital independent of other factors associated with this outcome. "Discrimination" measures a model's ability to distinguish between patients who did and did not die in hospital and was measured using the c-statistic [18]. "Calibration" measures the accuracy of a model's predicted risk of death and was measured by dividing the study cohort into deciles and strata based on the estimated risk of death. Within each decile and stratum, observed and expected death rates were deemed similar if the 95% confidence interval around the former (calculated using exact methods [19]) included the latter. Overall calibration was summarized using the Hosmer Lemeshow statistic [20]. Table cells containing less than five observations were censored to maintain patient confidentiality.

In the validation group, we then compared the predictive performance of models containing the KP-IRAM with and without the PIMR score. To do this, we used two statistical measures: the Integrated Discrimination Improvement (IDI) [21] and the Net Reclassification Improvement (NRI) [22]. The IDI is the discrimination slope (the mean predicted risk in patients with the event minus that of patients without the event) of a model with the KP-IRAM and PIMR as independent predictors minus the discrimination slope of a model with the KP-IRAM alone as the independent predictor. An IDI above zero indicates improved discrimination (i.e. a larger separation in mean predicted risk between events and nonevents) with the addition of the PIMR. The NRI represents the net proportion of correct reclassification (with correct reclassification defined as the predicted risk moving upwards for events and downwards for non-events) among events and non-events (calculated separately and then summed) when the predicted risk from the model with KP-IRAM and PIMR is compared to that from the model with KP-IRAM alone. We also calculated the net *number *of correct reclassifications when the PIMR was added to the KP-IRAM.

SAS 9.2 (Cary, NC) was used for all analyses. The study was approved by The Ottawa Hospital Research Ethics Board.

## Results

There were 369 588 admissions to The Ottawa Hospital between 1 April 2004 and 1 April 2009. 93 971 of these hospitalizations were excluded from this study because patients were less than 15 years of age (n = 36 820), patients were transferred from or to another hospital (n = 12 931), or admissions were obstetrical and delivery-related (n = 44 220). We excluded another 157 admissions because they were missing a primary condition group (required to calculate the KP-IRAM). This left a total of 275 460 hospital admissions (137 730 in both the derivation and the validation group) consisting of 172 396 unique individuals. A description of patients in the derivation cohort is provided in Table 2. The validation group did not differ significantly from the derivation group (see additional file 1).

**Table 2:** Description of study hospitalizations in derivation cohort

	Entire Cohort (n = 137 730)	Died in Hospital (n = 4 013)	Discharged Alive (n = 133 717)
Mean age (SD)	58.9 (18.4)	72.8 (14.6)	58.5 (18.3)

Female, n (%)	71 724 (52.1)	18.7 (46.5)	69 856 (52.2)

Urgent admission, n (%)	54 400 (39.5)	3 824 (95.3)	50 576 (37.8)

Surgical service, n (%)	89 586 (65.0)	821 (20.5)	88 765 (66.4)

Median Elixhauser score^16 ^(IQR)	0 (0-4)	10 (4-16)	0 (0-3)

Mean LAPS at admission* (SD)	11.6 (22.3)	52.4 (32.9)	10.4 (20.7)

Median risk of death at admission (IQR)**	0.0011 (0.0001-0.0135)	0.1896 (0.0869-0.3517)	0.0009 (0.0001-0.0109)

Most Common Procedures, n (%)			

Lens excision	18571 (13.5%)	0 (0.0%)	18571 (13.9%)

Angioplasty	4177 (3.0%)	68 (1.7%)	4109 (3.1%)

Pharmacotherapy, total body	3652 (2.7%)	93 (2.3%)	3559 (2.7%)

Respiratory ventilation	3236 (2.3%)	682 (17.0%)	2554 (1.9%)

Repair, muscles of the chest and abdomen	3169 (2.3%)	24 (0.6%)	3145 (2.4%)

Partial hysterectomy	2628 (1.9%)	0 (0.0%)	2628 (2.0%)

Installation of external appliance, circulatory system NEC	2514 (1.8%)	104 (2.6%)	2410 (1.8%)

Total excision of vitreous	2385 (1.7%)	0 (0.0%)	2385 (1.8%)

Pharmacotherapy (local), vessels of heart	2182 (1.6%)	26 (0.6%)	2156 (1.6%)

Total hysterectomy	2211-5 (1.6%)	< = 5 (0.0%)	2210 (1.7%)

*Laboratory Acute Physiology Score (LAPS). The LAPS is based on 14 laboratory test results obtained in the 24 hours prior to the index day. For people undergoing one of the procedures considered in the model (see additional file 2), the index day was the day of the procedure. For all others, the index day was the day of admission. Increasing degrees of physiologic derangement are reflected in a higher LAPS, which is a continuous variable that can range between a minimum of zero and a theoretical maximum of 256.

** Predicted using Kaiser-Permanente in-patient risk model [7].

In the entire cohort, a total of 1939 therapeutic procedures were coded during the study period. 1436 procedures were excluded because less than one procedure per month was performed during the study period. The remaining 503 procedures included a total of 938 procedure-urgency combinations. After adjusting for the Kaiser Permanente In-patient Risk Adjustment Model (KP-IRAM) death risk estimate, the p-value of the association of 726 of these procedure-urgency combinations exceeded 0.5 in the derivation cohort and were therefore excluded. This left a total of 212 procedure-urgency combinations (including 168 individual surgeries) expressed as binomial (i.e. 1/0) variables that were offered to the logistic model (see additional file 2).

After adjusting for important patient and admission factors, 56 procedure-urgency combinations (comprising 52 individual procedures) were independently associated with death in hospital (Table 3). 37 emergent and eight elective procedures were independently associated with an increased risk of death in hospital, while four emergent and seven elective procedures were protective. In the validation set, there were 22 664 (16.4%) admissions where the patient underwent at least one PIMR procedure, with 83% of these procedures occurring within the first three days of the hospitalization. Procedures having the strongest association with death in hospital included cardiac resuscitation, ventriculectomy, pericardial drainage, and pelvic irradiation. A full description of each procedure that was independently associated with death in hospital is given in Additional File 3.

**Table 3:** Procedures independently associated with death in hospital

Variable	Para-meter Estimate	Adjusted Odds Ratio	(95% CI)	PIMR Score
Predicted risk of death*	1.03	2.79	2.73 - 2.86	-

**Emergent Procedures**				

Resuscitation, heart NEC	4.26	70.72	41.04 - 121.84	11

Excision partial, ventricle	3.71	40.91	5.03 - 333.05	10

Repair, aortic valve	2.91	18.27	4.90 - 68.19	8

Immobilization, shoulder joint	2.95	19.18	1.55 - 236.57	8

Repair, patella	2.81	16.60	1.61 - 171.01	7

Repair, tricuspid valve	2.38	10.85	1.77 - 66.34	6

Implantation of device, descending thoracic aorta	2.28	9.82	5.73 - 16.84	6

Occlusion, abdominal arteries NEC	2.44	11.53	4.67 - 28.49	6

Implantation of internal device, abdominal cavity	2.43	11.33	2.21 - 58.17	6

Excision partial soft tissue, chest and abdomen	2.46	11.76	2.38 - 58.01	6

Drainage, ventricles of brain	1.88	6.54	3.26 - 13.11	5

Drainage, bronchus NEC	1.77	5.89	1.33 - 26.20	5

Ventilation, respiratory system NEC	1.89	6.65	5.73 - 7.71	5

Installation of external appliance, heart NEC	2.00	7.38	2.72 - 20.01	5

Stimulation, heart NEC	1.54	4.68	2.89 - 7.56	4

Extraction, arteries of leg NEC	1.47	4.34	2.38 - 7.93	4

Bypass, small intestine	1.49	4.43	1.95 - 10.04	4

Repair, small intestine	1.35	3.86	1.91 - 7.80	4

Drainage, meninges and dura mater of brain	1.23	3.43	2.15 - 5.48	3

Excision partial, brain	1.33	3.77	1.98 - 7.20	3

Control of bleeding, thoracic cavity NEC	1.06	2.90	1.37 - 6.12	3

Drainage, pericardium	1.01	2.74	1.18 - 6.37	3

Occlusion, vena cava (superior and inferior)	1.25	3.50	1.60 - 7.65	3

Control of bleeding, esophagus	1.14	3.12	1.46 - 6.71	3

Dilation, esophagus	1.09	2.98	1.18 - 7.54	3

Control of bleeding, small and large intestine	1.10	3.00	1.22 - 7.38	3

Amputation, tibia and fibula	1.03	2.80	1.27 - 6.21	3

Bypass with exteriorization, trachea	0.58	1.78	1.12 - 2.84	2

Implantation of internal device, stomach	0.61	1.84	1.25 - 2.70	2

Excision partial, small intestine	0.78	2.18	1.33 - 3.58	2

Excision partial, large intestine	0.58	1.79	1.20 - 2.66	2

Drainage, abdominal cavity	0.67	1.96	1.43 - 2.69	2

Implantation of internal device, hip joint	0.82	2.26	1.59 - 3.22	2

Fixation, femur	0.76	2.13	1.52 - 2.99	2

Amputation, femur	0.92	2.51	1.20 - 5.24	2

Drainage, pleura	0.56	1.74	1.36 - 2.24	1

Implantation of internal device, vena cava	0.38	1.46	1.20 - 1.78	1

Pharmacotherapy (local), vessels of heart	-0.68	0.51	0.31 - 0.85	-2

Excision total, appendix	-1.13	0.32	0.12 - 0.88	-3

Installation of external appliance, circulatory system NEC	-1.39	0.25	0.17 - 0.36	-4

Excision partial, abdominal cavity	-2.35	0.10	0.01 - 0.72	-6

**Elective Procedures**				

Drainage, pericardium	3.37	29.16	6.28 - 135.28	9

Radiation, pelvis	3.35	28.47	2.58 - 314.61	9

Destruction, skin of abdomen and trunk	2.75	15.61	1.89 - 128.67	7

Excision partial, cerebellum	2.17	8.75	2.01 - 38.11	6

Pharmacotherapy, circulatory system NEC	2.29	9.92	2.26 - 43.51	6

Repair, abdominal arteries NEC	1.75	5.77	1.36 - 24.54	5

Amputation, femur	1.92	6.81	1.93 - 24.01	5

Ventilation, respiratory system NEC	0.83	2.28	1.66 - 3.14	2

Dilation, coronary arteries	-1.61	0.20	0.06 - 0.69	-4

Implantation of internal device, hip joint	-1.57	0.21	0.07 - 0.65	-4

Implantation of internal device, knee joint	-1.58	0.21	0.08 - 0.55	-4

Excision total, ovary with fallopian tube	-2.07	0.13	0.02 - 0.91	-5

Repair, muscles of the chest and abdomen	-2.00	0.14	0.05 - 0.36	-5

Excision partial, prostate	-2.24	0.11	0.01 - 0.76	-6

Excision total, uterus and surrounding structures	-2.51	0.08	0.01 - 0.59	-7

*The adjusted odds ratio represents the increased risk for every 10% increase in death risk from the KP in-patient risk model.

See Additional File 3 for codes and a full description of each procedure (along with all component procedures).

NEC - Not elsewhere classified

Four procedures were independently associated with risk of death in hospital regardless of whether the procedure was done emergently or electively (Table 4). In two cases, the elective version of the procedure was assigned more points (indicating a higher risk of death in hospital) than the emergent version of the procedure.

**Table 4:** Procedures independently associated with risk of death in-hospital regardless of procedure urgency

Procedural Description	Points for emergent procedure	Points for elective procedure
Ventilation, respiratory system NEC	5	2
Drainage, pericardium	3	9
Implantation of internal device, hip joint	2	-4
Amputation, femur	2	5

**Figure 1:**
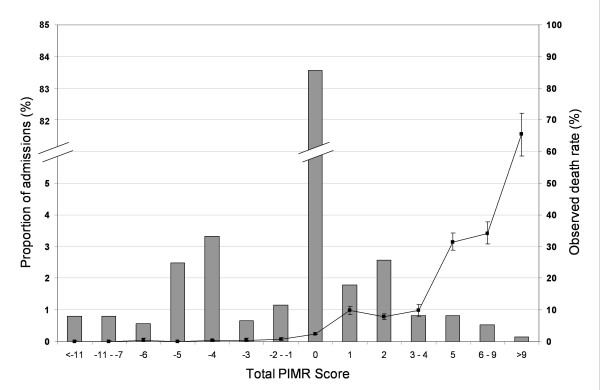
**Frequency distribution of the total Procedural Index for Mortality Risk (PIMR) score among validation admissions**. The horizontal axis presents the total PIMR score. The bars and left vertical axis presents the percent of hospitalizations with each total PIMR score. Individual PIMR scores were grouped to contain at least 0.5% of all admissions. The line and right vertical axis presents the observed number of deaths (with 95% confidence intervals) in each PIMR score.

Parameter estimates for procedures in the final logistic model were modified into the Procedural Index for Mortality Risk (PIMR) score (Table 3). The PIMR score for individual procedures ranged from -7 to +11. Since 84% of admissions had none of the included procedures, most hospitalizations had a total PIMR score of 0 (Figure 1, left axis). The risk of death in hospital was significantly associated with the PIMR score (Figure 1, right axis). By itself, the PIMR score was moderately discriminative for death in hospital (c-statistic 67.3%, 95% CI 66.6%-68.0%).

**Figure 2:**
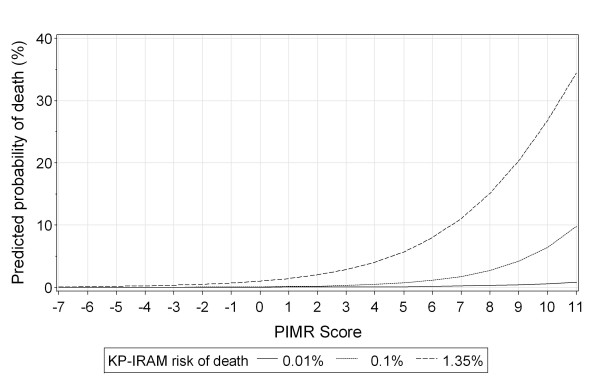
**Effect of adding the Procedural Index for Mortality Risk (PIMR) score to the Kaiser Permanente Inpatient Risk Adjustment Methodology (KP-IRAM) on predicted risk of death in hospital**. This graph presents the expected probability of death in hospital (vertical axis) for varying PIMR scores (horizontal axis). Risks are presented for people whose expected risk of death in hospital (based on the KP-IRAM) was at the 25^th ^percentile (0.01%, solid line), 50^th ^percentile (0.11%, long-dashed line), and 75^th ^percentile (1.35%, short-dashed line).

**Figure 3:**
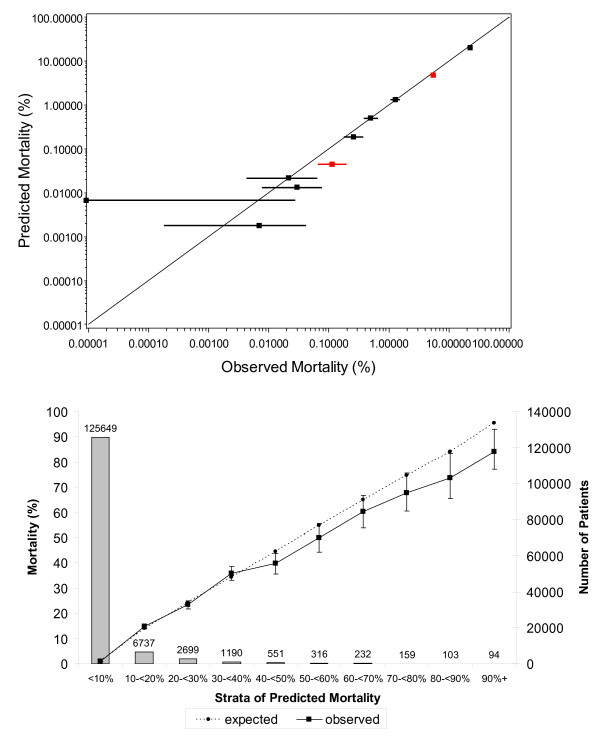
**Calibration of KP-IRAM and PIMR to predict death in hospital**. These figures compare observed and expected death rates when the validation group was divided into expected risk deciles (top) and strata (bottom). The decile plot presents observed mortality rates with 95% confidence intervals with those in red significantly differing from expected.

The total PIMR score significantly changed the expected risk of death in hospital beyond that estimated by the KP-IRAM (Figure 2). The total PIMR score also significantly improved the ability to predict risk of in-hospital death beyond that generated by the KP-IRAM. Model discrimination improved, as indicated by the c-statistic (increased from 0.929 [95% CI 0.926-0.932] to 0.938 [0.935-0.941]) and the Integrated Discrimination Improvement (IDI) (0.04327, 95% CI 0.0384-0.0482; *p *< .0001). Model calibration (Figure 3) did not change (Hosmer-Lemeshow fit statistic decreased from 37.56 to 36.51). The Net Reclassification Improvement (NRI) analysis showed that although the overall net *proportion *of correct reclassification was negative (-18.4%), the overall net *number *of correct reclassifications was positive (+17 923 or 13% of the entire cohort, Table 5).

**Table 5:** Results of the Net Reclassification Improvement (NRI) analysis:

Discharge status (N)	Correct reclassification*		**Incorrect reclassification**^†^		**Net correct reclassification**^‡^	
	
	%	N	%	N	%	N
Dead (4040)	33.6	1 357	66.4	2 683	-32.8	-1 325

Alive (133690)	57.2	76 471	42.8	57 219	+14.4	+19 251

**Overall net correct reclassification**					**-18.4**	**+17 926**

*Correct reclassification: 'up' for events and 'down' for non-events, where 'up' is a higher predicted risk of death from the new vs old model and 'down' is a lower predicted risk of death from the new vs old model

^†^Incorrect reclassification: 'down' for events and 'up' for non-events

^‡^Net correct reclassification: correct reclassification - incorrect reclassification

## Discussion

We derived and internally validated an index that used administrative data to quantify the relative contribution of a broad range of therapeutic procedures on the risk of death in hospital. We identified 52 procedures which (after adjusting for a robust and validated hospital mortality model) were significantly associated with the risk of death in hospital. We modified this model into an index that reflects the independent contribution of each procedure to the risk of death in hospital. By itself, and when added to an accurate model to predict hospital mortality, the total Procedural Index for Mortality Risk (PIMR) score significantly predicted risk of death in hospital.

The importance of surgical interventions on hospital outcomes is reflected by the large number of indexes that use patient and hospitalization factors to predict the risk of post-procedural death (Table 1) [1-6]. The clinical variables in these indexes, along with their simplicity, increase their face validity to practicing clinicians. However, these clinical variables prohibit calculation of these indexes using administrative data. To develop our index, we started with a validated, highly accurate model to predict hospital mortality risk in all hospital patients. We then determined the risk of death after a broad range of procedures *independent *of that predicted from the KP-IRAM. Both by itself and when added to the KP-IRAM model, the PIMR was significantly associated with the risk of death in hospital.

The PIMR would primarily be used in analyses involving administrative data. Expressing this risk as a simple score facilitates our understanding of the relative importance of various interventions on death in hospital. When combined with the KP-IRAM, the PIMR had excellent discrimination and calibration for predicting risk of death in hospital. It is notable that the discrimination achieved with the KP-IRAM and PIMR was similar to that achieved using clinical based models (Table 1). The PIMR will allow researchers and administrators to gauge patient and procedural complexity of individual surgeons, services, or hospitals for descriptive or comparative purposes and will also let analysts adjust for the influence of a large range of therapeutic procedures on risk of hospital mortality.

The independent association between many of the PIMR procedures and risk of hospital death may reflect unresolved confounding of patient or hospitalization factors. The significance of several procedures (e.g. cardiac resuscitation) is likely due to important clinical events (e.g. cardiac arrest) that are identified by the procedure code and are not captured by the KP-IRAM. Further work is required to determine how much mortality risk is due to the procedure and how much is due to other underlying patient factors.

The addition of the PIMR to the KP-IRAM model significantly improved the ability to predict hospital mortality. The absolute increase of the model's c-statistic was small (0.009 or 0.9%). Several studies have shown that the overall, sequential improvement of model performance decreases as more and more variables are added [23,24]. However, the c-statistic of the KP-IRAM was already very high without the PIMR score (92.9%). With the PIMR, the C-statistic increased more than 10% of the distance between the KP-IRAM and perfect discrimination. This indicates, along with the results presented in Figure 1, the strength of PIMR to predict risk of death with or without other covariates associated with death risk in hospital.

We believe that two steps could greatly improve the PIMR. The PIMR relies on procedure codes whose accuracy has not been validated. Our study's objective was to derive and validate an index that determines the independent influence of various procedures on hospital mortality. Strictly speaking, however, the PIMR measures the independent influence of *codes *for various procedures - rather than the procedures themselves - on hospital mortality. Without knowing the accuracy of each code for its respective procedure, we are uncertain how strong a surrogate each code is for the actual procedure. Before one uses the PIMR for individual patient risk prediction, the accuracy of the procedure codes contained in the PIMR should be validated.

The second major limitation of the PIMR is its imputation of procedural urgency using admission urgency status. For most hospitalizations, admission and procedural urgency will be identical but situations could arise in which they would differ. For example, consider a patient admitted electively for a hip replacement who has an acute myocardial infarction requiring an emergent angioplasty. In this case, the angioplasty urgency would be misclassified as an elective procedure. We believe that this bias explains why the number of points assigned to two elective procedures exceeded that for their emergent counterpart (Table 4). The PIMR could be improved by using an accurate classification of procedural urgency.

There are other limitations to the PIMR. First, the PIMR requires that surgical procedures are coded using the Canadian Classification of Interventions (CCI). Without validated translation tables to other procedural coding systems, this limits its use to Canadian institutions. Second, the PIMR was derived and validated in a single hospital. While objective and universal criteria are used to code procedures, it is possible that local coding practices could change the PIMR's validity in other patient populations. Third, most procedures are not included in the PIMR because they were not independently associated with risk of death in hospital. As a result, the PIMR should be used as an adjunct to other factors associated with risk of death in hospital - such as those in the KP-IRAM - to compare outcomes after various surgeries. Researchers should exercise some caution if this index is used when inferring institutional quality of care issues using hospital mortality. Some of the components in the PIMR (such as heart resuscitation) could result from poor quality of care, the adjustment of which could hide such problems.

Finally, our analyses did not include surgeries that were infrequently conducted at our hospital.

## Conclusion

We have derived and internally validated an index to express the independent association of a broad range of procedures with risk of death in hospital. When this is added to a validated hospital death risk index, we can accurately predict post-procedural risk of death.

## Competing interests

The authors declare that they have no competing interests.

## Authors' contributions

CvW conceived of the study; directed the study design and statistical analysis; and drafted the manuscript. JW participated in the study design; performed the statistical analysis; created the tables, additional files, and figures; and edited the manuscript. CB performed the literature search and extracted the information in Table 1. AF provided the study data, participated in the study design, and reviewed the manuscript for important clinical and intellectual content. All authors have read and approved the final manuscript.

## Pre-publication history

The pre-publication history for this paper can be accessed here:

http://www.biomedcentral.com/1472-6963/11/258/prepub

## Supplementary Material

Additional file 1 **Comparison of study derivation and validation cohort**. Additional file 1 contains descriptive statistics of the derivation and validation cohort. Click here for file

Additional file 2 **List of the 212 unique procedure-urgency combinations offered to the multivariate logistic model**. Additional file 2 contains the frequency (in the derivation set), description, and 5-digit CCI code of the 212 procedure-urgency combinations that were offered to the multivariate logistic model. The p-value for the association of each of these 212 procedure-urgency combinations with death in hospital was < 0.5 (after adjusting for the risk of death in-hospital, as measured with KP-IRAM). Click here for file

Additional file 3 **Full description of all procedure-urgency combinations independently associated with in-hospital death**. Additional file 3 contains the frequency and *full *CCI code and description of all procedure-urgency combinations independently associated with in-hospital death (i.e. included in the PIMR index), as observed in the derivation set. Click here for file
