# Supplementary material for: The Procedural Index for Mortality Risk (PIMR): an index calculated using administrative data to quantify the independent influence of procedures on risk of hospital death
Source: BMC Health Serv Res. 2011 Oct 7;11:258. doi: 10.1186/1472-6963-11-258 (PMC3200180; doi:10.1186/1472-6963-11-258)
Supplement: Additional file 1 — Comparison of study derivation and validation cohort. Additional file 1 contains descriptive statistics of the derivation and validation cohort. [file 1472-6963-11-258-S1.DOC]

**ADDITIONAL FILE 1 - Comparison of study derivation and validation cohort.**

|  | **Derivation Cohort**  (n=137 730) | **Validation Cohort**  (n=137 730) |
| --- | --- | --- |
| Mean age (SD) | 58.9 (18.4) | 58.8 (18.3) |
| Female, n (%) | 71 724 (52.1) | 71 445 (51.9) |
| Urgent admission, n (%) | 54 400 (39.5) | 54 171 (39.3) |
| Surgical service, n (%) | 89 586 (65.0) | 89 869 (65.3) |
| Median Elixhauser score[16] (IQR) | 0 (0-4) | 0 (0-4) |
| Mean LAPS at admission* (SD) | 11.6 (22.3) | 11.5 (22.4) |
| Median risk of death at admission (IQR)** | 0.0011 (0.0001-0.0135) | 0.0011 (0.0001-0.0131) |
| Most Common Procedures, n (%) |  |  |
| Lens excision | 18 571 (13.5) | 18 335 (13.3) |
| Angioplasty | 4177 (3.0) | 4198 (3.0) |
| Pharmacotherapy, total body | 3652 (2.7) | 3674 (2.7) |
| Respiratory ventilation | 3236 (2.3) | 3293 (2.4) |
| Repair, muscles of the chest and abdomen | 3169 (2.3) | 3231 (2.3) |
| Partial hysterectomy | 2628 (1.9) | 2607 (1.9) |
| Installation of external appliance, circulatory system NEC | 2514 (1.8) | 2611 (1.9) |
| Total excision of vitreous | 2385 (1.7) | 2404 (1.7) |
| Pharmacotherapy (local), vessels of heart | 2182 (1.6) | 2146 (1.6) |
| Total hysterectomy | 2211-5 (1.6) | 2170 (1.6) |

*Laboratory Acute Physiology Score (LAPS). The LAPS is based on 14 laboratory test results obtained in the 24 hours preceding hospitalization. Increasing degrees of physiologic derangement are reflected in a higher LAPS, which is a continuous variable that can range between a minimum of zero and a theoretical maximum of 256.

** Predicted using Kaiser-Permanente in-patient risk model [7].
