# Supplementary material for: The Procedural Index for Mortality Risk (PIMR): an index calculated using administrative data to quantify the independent influence of procedures on risk of hospital death
Source: BMC Health Serv Res. 2011 Oct 7;11:258. doi: 10.1186/1472-6963-11-258 (PMC3200180; doi:10.1186/1472-6963-11-258)
Supplement: Additional file 2 — List of the 212 unique procedure-urgency combinations offered to the multivariate logistic model. Additional file 2 contains the frequency (in the derivation set), description, and 5-digit CCI code of the 212 procedure-urgency combinations that were offered to the multivariate logistic model. The p-value for the association of each of these 212 procedure-urgency combinations with death in hospital was < 0.5 (after adjusting for the risk of death in-hospital, as measured with KP-IRAM). [file 1472-6963-11-258-S2.DOC]

**ADDITIONAL FILE 2 - List of the 212 unique procedure-urgency combinations offered to the multivariate logistic model**

| **5-digit CCI code** | **Description** | **Procedure Urgency** | **# of admissions in derivation set** |
| --- | --- | --- | --- |
| 1AA52 | Drainage, meninges and dura mater of brain | Emergent | 173 |
| 1AA80 | Repair, meninges and dura mater of brain | Emergent | 53 |
| 1AC52 | Drainage, ventricles of brain | Emergent | 67 |
| 1AC54 | Management of internal device, ventricles of brain | Emergent | 35 |
| 1AJ87 | Excision partial, cerebellum | Emergent | 16 |
| 1AJ87 | Excision partial, cerebellum | Elective | 20 |
| 1AN27 | Radiation, brain | Emergent | 140 |
| 1AN27 | Radiation, brain | Elective | 9 |
| 1AN52 | Drainage, brain | Emergent | 37 |
| 1AN87 | Excision partial, brain | Emergent | 135 |
| 1AW72 | Release, spinal cord | Emergent | 120 |
| 1AW72 | Release, spinal cord | Elective | 431 |
| 1AX35 | Pharmacotherapy (local). spinal canal and meninges | Emergent | 173 |
| 1AX35 | Pharmacotherapy (local). spinal canal and meninges | Elective | 530 |
| 1BM72 | Release, nerve(s) of upper arm and elbow | Emergent | 24 |
| 1EA80 | Repair, cranium | Emergent | 11 |
| 1ET73 | Reduction, nose | Emergent | 8 |
| 1EY87 | Excision partial, paranasal sinuses | Emergent | 10 |
| 1FD80 | Repair, gingiva | Elective | 213 |
| 1FE57 | Extraction, tooth | Elective | 289 |
| 1FU89 | Excision total, thyroid gland | Elective | 242 |
| 1GE91 | Excision radical, larynx NEC | Elective | 36 |
| 1GJ77 | Bypass with exteriorization, trachea | Emergent | 175 |
| 1GJ77 | Bypass with exteriorization, trachea | Elective | 126 |
| 1GM52 | Drainage, bronchus NEC | Emergent | 18 |
| 1GR87 | Excision partial, lobe of lung | Elective | 197 |
| 1GT27 | Radiation, lung NEC | Emergent | 126 |
| 1GV52 | Drainage, pleura | Emergent | 826 |
| 1GV87 | Excision partial, pleura | Elective | 81 |
| 1GX80 | Repair, diaphragm | Emergent | 21 |
| 1GY13 | Control of bleeding, thoracic cavity NEC | Emergent | 93 |
| 1GY72 | Release, thoracic cavity NEC | Emergent | 7 |
| 1GY72 | Release, thoracic cavity NEC | Elective | 31 |
| 1GZ31 | Ventilation, respiratory system NEC | Emergent | 2102 |
| 1GZ31 | Ventilation, respiratory system NEC | Elective | 1134 |
| 1HA52 | Drainage, pericardium | Emergent | 58 |
| 1HA52 | Drainage, pericardium | Elective | 9 |
| 1HD53 | Implantation of internal device, endocardium | Emergent | 19 |
| 1HH59 | Destruction, cardiac conduction system | Emergent | 143 |
| 1HH59 | Destruction, cardiac conduction system | Elective | 298 |
| 1HN80 | Repair, interatrial septum | Emergent | 35 |
| 1HN80 | Repair, interatrial septum | Elective | 181 |
| 1HP87 | Excision partial, ventricle | Emergent | 5 |
| 1HS80 | Repair, tricuspid valve | Emergent | 13 |
| 1HU80 | Repair, mitral valve | Elective | 229 |
| 1HU90 | Excision total with reconstruction, mitral valve | Emergent | 35 |
| 1HU90 | Excision total with reconstruction, mitral valve | Elective | 88 |
| 1HV80 | Repair, aortic valve | Emergent | 13 |
| 1HV90 | Excision total with reconstruction, aortic valve | Emergent | 167 |
| 1HV90 | Excision total with reconstruction, aortic valve | Elective | 418 |
| 1HZ09 | Stimulation, heart NEC | Emergent | 190 |
| 1HZ09 | Stimulation, heart NEC | Elective | 105 |
| 1HZ30 | Resuscitation, heart NEC | Emergent | 127 |

**Appendix B (continued).** A list of the 212 unique procedure-urgency combinations offered to the multivariate logistic model

| **5-digit CCI code** | **Description** | **Procedure Urgency** | **# of admissions in derivation set** |
| --- | --- | --- | --- |
| 1HZ37 | Installation of external appliance, heart NEC | Emergent | 41 |
| 1HZ53 | Implantation of internal device, heart NEC | Emergent | 507 |
| 1HZ55 | Removal of device, heart NEC | Emergent | 27 |
| 1IA80 | Repair, ascending aorta | Elective | 17 |
| 1IA87 | Excision partial, ascending aorta | Emergent | 23 |
| 1IA87 | Excision partial, ascending aorta | Elective | 35 |
| 1IC53 | Implantation of internal device, thoracic [descending] aorta | Emergent | 100 |
| 1ID80 | Repair, aorta NEC | Elective | 35 |
| 1IJ50 | Dilation, coronary arteries | Elective | 971 |
| 1IJ76 | Bypass, coronary arteries | Emergent | 799 |
| 1IL35 | Pharmacotherapy (local), vessels of heart | Emergent | 1575 |
| 1IL35 | Pharmacotherapy (local), vessels of heart | Elective | 607 |
| 1IM57 | Extraction, pulmonary artery | Emergent | 13 |
| 1IM57 | Extraction, pulmonary artery | Elective | 17 |
| 1IS51 | Occlusion, vena cava (superior and inferior) | Emergent | 60 |
| 1IS51 | Occlusion, vena cava (superior and inferior) | Elective | 2 |
| 1IS53 | Implantation of internal device, vena cava (superior and inferior) | Emergent | 1523 |
| 1IS55 | Removal of device, vena cava (superior and inferior) | Emergent | 35 |
| 1JE50 | Dilation, carotid artery | Emergent | 10 |
| 1JE57 | Extraction, carotid artery | Elective | 213 |
| 1JM57 | Extraction, arteries of arm NEC | Emergent | 30 |
| 1JM76 | Bypass, arteries of arm NEC | Emergent | 38 |
| 1JU58 | Procurement, veins of arm NEC | Emergent | 15 |
| 1JU58 | Procurement, veins of arm NEC | Elective | 26 |
| 1KA76 | Bypass, abdominal aorta | Emergent | 19 |
| 1KA76 | Bypass, abdominal aorta | Elective | 90 |
| 1KA80 | Repair, abdominal aorta | Emergent | 81 |
| 1KE51 | Occlusion, abdominal arteries NEC | Emergent | 32 |
| 1KE51 | Occlusion, abdominal arteries NEC | Elective | 59 |
| 1KE76 | Bypass, abdominal arteries | Emergent | 17 |
| 1KE76 | Bypass, abdominal arteries | Elective | 35 |
| 1KE80 | Repair, abdominal arteries NEC | Emergent | 14 |
| 1KE80 | Repair, abdominal arteries NEC | Elective | 29 |
| 1KG50 | Dilation, arteries of leg NEC | Emergent | 55 |
| 1KG51 | Occlusion, arteries of leg NEC | Elective | 19 |
| 1KG57 | Extraction, arteries of leg NEC | Emergent | 135 |
| 1KG57 | Extraction, arteries of leg NEC | Elective | 104 |
| 1KG76 | Bypass, arteries of leg NEC | Emergent | 205 |
| 1KG80 | Repair, arteries of leg NEC | Emergent | 45 |
| 1KR58 | Procurement, veins of leg NEC | Emergent | 529 |
| 1KV53 | Implantation of internal device, artery NEC | Emergent | 29 |
| 1KV53 | Implantation of internal device, artery NEC | Elective | 29 |
| 1KX53 | Implantation of internal device, vein NEC | Emergent | 31 |
| 1LZ19 | Transfusion, circulatory system NEC | Emergent | 43 |
| 1LZ19 | Transfusion, circulatory system NEC | Elective | 223 |
| 1LZ35 | Pharmacotherapy (local), circulatory system NEC | Elective | 19 |
| 1LZ37 | Installation of external appliance, circulatory system NEC | Emergent | 940 |
| 1LZ37 | Installation of external appliance, circulatory system NEC | Elective | 1574 |
| 1MH87 | Excision partial, lymph node(s), pelvic | Emergent | 7 |
| 1MH87 | Excision partial, lymph node(s), pelvic | Elective | 174 |
| 1MH89 | Excision total, lymph node(s), pelvic | Emergent | 7 |
| 1MH89 | Excision total, lymph node(s), pelvic | Elective | 214 |

**Appendix B (continued).** A list of the 212 unique procedure-urgency combinations offered to the multivariate logistic model

| **5-digit CCI code** | **Description** | **Procedure Urgency** | **# of admissions in derivation set** |
| --- | --- | --- | --- |
| 1NA13 | Control of bleeding, esophagus | Emergent | 61 |
| 1NA50 | Dilation, esophagus | Emergent | 60 |
| 1NA80 | Repair, esophagus | Emergent | 20 |
| 1NA87 | Excision partial, esophagus | Emergent | 3 |
| 1NE72 | Release, pylorus | Elective | 36 |
| 1NF13 | Control of bleeding, stomach | Emergent | 101 |
| 1NF53 | Implantation of internal device, stomach | Emergent | 272 |
| 1NF87 | Excision partial, stomach | Emergent | 25 |
| 1NK53 | Implantation of internal device, small intestine | Elective | 56 |
| 1NK58 | Procurement, small intestine | Elective | 62 |
| 1NK76 | Bypass, small intestine | Emergent | 48 |
| 1NK77 | Bypass with exteriorization, small intestine | Emergent | 77 |
| 1NK77 | Bypass with exteriorization, small intestine | Elective | 154 |
| 1NK80 | Repair, small intestine | Emergent | 86 |
| 1NK82 | Reattachment, small intestine | Emergent | 16 |
| 1NK87 | Excision partial, small intestine | Emergent | 262 |
| 1NK87 | Excision partial, small intestine | Elective | 155 |
| 1NM77 | Bypass with exteriorization, large intestine | Emergent | 71 |
| 1NM80 | Repair, large intestine | Emergent | 23 |
| 1NM87 | Excision partial, large intestine | Emergent | 401 |
| 1NM87 | Excision partial, large intestine | Elective | 537 |
| 1NM89 | Excision total, large intestine | Emergent | 31 |
| 1NP13 | Control of bleeding, small and large intestine | Emergent | 78 |
| 1NP72 | Release, small and large intestine | Emergent | 107 |
| 1NP73 | Reduction, small and large intestine | Emergent | 21 |
| 1NP86 | Closure of fistula, small and large intestine | Emergent | 17 |
| 1NQ52 | Drainage, rectum | Emergent | 40 |
| 1NQ87 | Excision partial, rectum | Elective | 373 |
| 1NQ89 | Excision total, rectum | Emergent | 11 |
| 1NV89 | Excision total, appendix | Emergent | 948 |
| 1OA52 | Drainage, liver | Emergent | 26 |
| 1OB89 | Excision total, spleen | Emergent | 38 |
| 1OD52 | Drainage, gallbladder | Emergent | 53 |
| 1OE50 | Dilation, bile ducts | Emergent | 96 |
| 1OE52 | Drainage, bile ducts | Elective | 17 |
| 1OE57 | Extraction, bile ducts | Emergent | 162 |
| 1OE76 | Bypass, bile ducts | Emergent | 8 |
| 1OJ87 | Excision partial, pancreas | Elective | 34 |
| 1OT52 | Drainage, abdominal cavity | Emergent | 572 |
| 1OT53 | Implantation of internal device, abdominal cavity | Emergent | 11 |
| 1OT72 | Release, abdominal cavity | Emergent | 218 |
| 1OT72 | Release, abdominal cavity | Elective | 827 |
| 1OT87 | Excision partial, abdominal cavity | Emergent | 62 |
| 1OT87 | Excision partial, abdominal cavity | Elective | 473 |
| 1OW80 | Repair, surgically constructed sites in digestive & biliary tract | Emergent | 16 |
| 1PB89 | Excision total, adrenal gland | Emergent | 4 |
| 1PE50 | Dilation, renal pelvis | Emergent | 366 |
| 1PE52 | Drainage, renal pelvis | Emergent | 160 |
| 1PG50 | Dilation, ureter NEC | Emergent | 167 |
| 1PG50 | Dilation, ureter NEC | Elective | 173 |
| 1PM57 | Extraction, bladder NEC | Elective | 158 |
| 1PM87 | Excision partial, bladder NEC | Emergent | 95 |
| 1PM92 | Excision radical with reconstruction, bladder NEC | Emergent | 7 |

**Appendix B (continued).** A list of the 212 unique procedure-urgency combinations offered to the multivariate logistic model

| **5-digit CCI code** | **Description** | **Procedure Urgency** | **# of admissions in derivation set** |
| --- | --- | --- | --- |
| 1PM92 | Excision radical with reconstruction, bladder NEC | Elective | 70 |
| 1PQ50 | Dilation, urethra NEC | Emergent | 24 |
| 1QM89 | Excision total, testis | Emergent | 18 |
| 1QT87 | Excision partial, prostate | Emergent | 45 |
| 1QT87 | Excision partial, prostate | Elective | 772 |
| 1RD89 | Excision total, ovary with fallopian tube | Emergent | 73 |
| 1RD89 | Excision total, ovary with fallopian tube | Elective | 1770 |
| 1RM89 | Excision total, uterus and surrounding structures | Elective | 2181 |
| 1RW87 | Excision partial, vulva NEC | Emergent | 4 |
| 1SC27 | Radiation, spinal vertebrae | Emergent | 101 |
| 1SC75 | Fusion, spinal vertebrae | Emergent | 146 |
| 1SC80 | Repair, spinal vertebrae | Elective | 479 |
| 1SQ27 | Radiation, pelvis | Emergent | 24 |
| 1SQ27 | Radiation, pelvis | Elective | 3 |
| 1SQ58 | Procurement, pelvis | Emergent | 94 |
| 1SQ58 | Procurement, pelvis | Elective | 418 |
| 1SY55 | Removal of device, muscles of chest and abdomen | Emergent | 21 |
| 1SY58 | Procurement, muscles of the chest and abdomen | Elective | 113 |
| 1SY80 | Repair, muscles of the chest and abdomen | Emergent | 356 |
| 1SY80 | Repair, muscles of the chest and abdomen | Elective | 2813 |
| 1SZ87 | Excision partial, soft tissue of the chest and abdomen | Emergent | 10 |
| 1TA03 | Immobilization, shoulder joint | Emergent | 3 |
| 1TA87 | Excision partial, shoulder joint | Elective | 160 |
| 1TC80 | Repair, rotator cuff | Elective | 455 |
| 1TK74 | Fixation, humerus | Elective | 24 |
| 1VA53 | Implantation of internal device, hip joint | Emergent | 568 |
| 1VA53 | Implantation of internal device, hip joint | Elective | 1384 |
| 1VA87 | Excision partial, hip joint | Emergent | 34 |
| 1VC55 | Removal of device, femur | Emergent | 35 |
| 1VC74 | Fixation, femur | Emergent | 683 |
| 1VC93 | Amputation, femur | Emergent | 86 |
| 1VC93 | Amputation, femur | Elective | 28 |
| 1VG35 | Pharmacotherapy (local), knee joint | Emergent | 29 |
| 1VG53 | Implantation of internal device, knee joint | Elective | 1809 |
| 1VP80 | Repair, patella | Emergent | 4 |
| 1VQ93 | Amputation, tibia and fibula | Emergent | 86 |
| 1VQ93 | Amputation, tibia and fibula | Elective | 32 |
| 1VX59 | Destruction, soft tissue of leg | Emergent | 91 |
| 1VX59 | Destruction, soft tissue of leg | Elective | 23 |
| 1VX87 | Excision partial, soft tissue of leg | Emergent | 7 |
| 1WB80 | Repair, foot ligaments | Emergent | 17 |
| 1WJ87 | Excision partial, tarsometatarsal joints, metatarsal bones and metatarsophalangeal joints [forefoot] | Emergent | 20 |
| 1WJ93 | Amputation, tarsometatarsal joints, metatarsal bones and metatarsophalangeal joints [forefoot] | Emergent | 126 |
| 1WZ27 | Radiation, musculoskeletal system NEC | Emergent | 49 |
| 1YF80 | Repair, skin of face | Emergent | 25 |
| 1YS52 | Drainage, skin of abdomen and trunk | Emergent | 16 |
| 1YS59 | Destruction, skin of abdomen and trunk | Elective | 8 |
| 1YS87 | Excision partial, skin of abdomen and trunk | Emergent | 4 |
| 1YT80 | Repair, skin of arm | Emergent | 18 |
| 1YU80 | Repair, skin of hand | Emergent | 18 |
| 1YV58 | Procurement, skin of leg | Emergent | 39 |

**Appendix B (continued).** A list of the 212 unique procedure-urgency combinations offered to the multivariate logistic model

| **5-digit CCI code** | **Description** | **Procedure Urgency** | **# of admissions in derivation set** |
| --- | --- | --- | --- |
| 1YV80 | Repair, skin of leg | Emergent | 21 |
| 1YV87 | Excision partial, skin of leg | Emergent | 3 |
| 1ZZ35 | Pharmacotherapy, total body | Elective | 2445 |
