# Supplementary material for: The Procedural Index for Mortality Risk (PIMR): an index calculated using administrative data to quantify the independent influence of procedures on risk of hospital death
Source: BMC Health Serv Res. 2011 Oct 7;11:258. doi: 10.1186/1472-6963-11-258 (PMC3200180; doi:10.1186/1472-6963-11-258)
Supplement: Additional file 3 — Full description of all procedure-urgency combinations independently associated with in-hospital death. Additional file 3 contains the frequency and full CCI code and description of all procedure-urgency combinations independently associated with in-hospital death (i.e. included in the PIMR index), as observed in the derivation set. [file 1472-6963-11-258-S3.DOC]

**ADDITIONAL FILE 3- Full description of all procedure-urgency combinations independently associated with in-hospital death**

| CCI code | Description | Procedure Urgency | Frequency in derivation set | PIMR score |
| --- | --- | --- | --- | --- |
| 1HZ30 | Resuscitation, heart NEC | Emergent | 128 | 11 |
| 1HZ30JN | with external manual compression |  | 54 |  |
| 1HZ30JY | with external manual compression and ventilation |  | 74 |  |
| 1HP87 | Excision partial, ventricle | Emergent | 5 | 10 |
| 1HP87LA | no tissue used [e.g. suture alone], open approach |  | 2 |  |
| 1HP87LAXXN | using synthetic material and open approach |  | 3 |  |
| 1HA52 | Drainage, pericardium | Elective | 10 | 9 |
| 1HA52HA | using percutaneous (needle) approach |  | 6 |  |
| 1HA52HATS | using percutaneous (needle) approach leaving drainage tube [catheter] in situ |  | 3 |  |
| 1HA52QB | using thoracic approach (e.g. sternotomy, thoracotomy) |  | 1 |  |
| 1SQ27 | Radiation, pelvis | Elective | 3 | 9 |
| 1SQ27JA | using external beam |  | 3 |  |
| 1HV80 | Repair, aortic valve | Emergent | 13 | 8 |
| 1HV80GPBD | using balloon (e.g. balloon valvuloplasty) percutaneous transluminal approach |  | 1 |  |
| 1HV80LA | using suture (e.g. annular plication, semicircular annuloplasty) open approach |  | 7 |  |
| 1HV80LAFE | using prosthetic ring (e.g. ring annuloplasty) open approach |  | 1 |  |
| 1HV80LAXXA | using autograft (e.g. patching of leaflet with pericardial tissue) open approach |  | 1 |  |
| 1HV80STBP | using dilation device (e.g. expanding dilator) closed heart technique [transventricular] |  | 3 |  |
| 1TA03 | Immobilization, shoulder joint | Emergent | 3 | 8 |
| 1TA03JASQ | using sling |  | 2 |  |
| 1TA03JASR | using splinting device [modified shoulder spica] |  | 1 |  |
| 1VP80 | Repair, patella | Emergent | 4 | 7 |
| 1VP80LA | using open approach and simple repair (for tissue regeneration) |  | 3 |  |
| 1VP80LAXXA | using open approach and autograft [e.g. bone, interpositional fascia] |  | 1 |  |
| 1YS59 | Destruction, skin of abdomen and trunk | Elective | 8 | 7 |
| 1YS59JAAG | using laser NEC [e.g. carbon dioxide for ablation] |  | 1 |  |
| 1YS59JACF | using mechanical device [sandpaper, wire brush] |  | 1 |  |
| 1YS59JAGX | using device NEC [electrocautery] |  | 6 |  |
| 1AJ87 | Excision partial, cerebellum | Elective | 20 | 6 |
| 1AJ87DAGX | endoscopic approach with device NEC |  | 1 |  |
| 1AJ87SZAZ | open approach with ultrasonic aspirator [e.g. CUSA] |  | 5 |  |
| 1AJ87SZGX | open approach with device NEC |  | 14 |  |
| 1HS80 | Repair, tricuspid valve | Emergent | 13 | 6 |
| 1HS80LA | using suture [e.g. annular plication, semicircular annuloplasty] open approach |  | 4 |  |
| 1HS80LAFE | using prosthetic ring [e.g. ring annuloplasty] open approach |  | 8 |  |
| 1HS80LAXXA | using autograft [e.g. patching of leaflet with pericardial tissue] open approach |  | 1 |  |
| 1IC53 | Implantation of internal device, thoracic [descending] aorta | Emergent | 100 | 6 |
| 1IC53GQQL | of intra-aortic balloon using percutaneous transluminal approach [e.g. through femoral artery] |  | 90 |  |
| 1IC53LAQL | of intra-aortic balloon using open approach |  | 10 |  |
| 1KE51 | Occlusion, abdominal arteries NEC | Emergent | 32 | 6 |
| 1KE51GQGE | percutaneous transluminal approach using (detachable) coil |  | 14 |  |
| 1KE51GQM0 | percutaneous transluminal approach and antineoplastic or immunomodulating agent |  | 1 |  |
| 1KE51GQW0 | percutaneous transluminal approach using synthetic agent [e.g. gelfoam, silicone, microspheres, polystyrene, polyvinyl alcohol] |  | 3 |  |
| 1KE51GQW3 | percutaneous transluminal approach using fibrin glue |  | 3 |  |
| 1KE51LA | open approach(e.g. arteriotomy) using direct suture |  | 10 |  |
| 1KE51LAFF | open approach(e.g. arteriotomy) using band or clip |  | 1 |  |
| 1LZ35 | Pharmacotherapy (local), circulatory system NEC | Elective | 19 | 6 |
| 1LZ35HHC1 | percutaneous infusion approach of antithrombotic agent |  | 5 |  |
| 1LZ35HHC6 | percutaneous infusion approach of parenteral nutrition |  | 12 |  |
| 1LZ35HHE0 | percutaneous infusion approach of cardiovascular system agent |  | 1 |  |
| 1LZ35HHZ9 | percutaneous infusion approach of agent NEC |  | 1 |  |
| 1OT53 | Implantation of internal device, abdominal cavity | Emergent | 11 | 6 |
| 1OT53DATS | of catheter (peritoneal dialysis) using endoscopic (laparoscopic) approach |  | 6 |  |
| 1OT53HATS | of catheter (peritoneal dialysis) using percutaneous (incision) approach |  | 4 |  |
| 1OT53LATS | of catheter (peritoneal dialysis) using open (laparotomy) approach |  | 1 |  |
| 1SZ87 | Excision partial, soft tissue of the chest and abdomen | Emergent | 10 | 6 |
| 1SZ87LA | using open approach and apposition [suture, staple] (to close surgical defect) |  | 8 |  |
| 1SZ87LAXXA | using open approach and autograft [e.g. fascia or skin] (to close surgical defect) |  | 1 |  |
| 1SZ87LAXXN | using open approach and synthetic tissue [e.g. mesh] (to close surgical defect) |  | 1 |  |
| 1AC52 | Drainage, ventricles of brain | Emergent | 67 | 5 |
| 1AC52DA | endoscopic [ventriculoscopic] approach drainage without shunt or catheter left in situ |  | 9 |  |
| 1AC52HA | percutaneous (via needle through fontanel) approach without shunt or catheter left in situ |  | 6 |  |
| 1AC52MBSJ | burr hole approach, shunt or catheter terminating at skin (of head) for temporary drainage |  | 24 |  |
| 1AC52MESJ | craniotomy or craniectomy [flap] approach shunt terminating in abdominal cavity [e.g. ventriculoperitoneal or ventriculobiliary] |  | 9 |  |
| 1AC52MPSJ | craniotomy or craniectomy [flap] approach shunt terminating in other site |  | 2 |  |
| 1AC52SESJ | burr hole approach, shunt terminating in abdominal cavity [e.g. ventriculoperitoneal or ventriculobiliary] |  | 13 |  |
| 1AC52SZ | craniotomy or craniectomy [flap] approach drainage without shunt or catheter left in situ |  | 4 |  |
| 1GM52 | Drainage, bronchus NEC | Emergent | 18 | 5 |
| 1GM52BATJ | using endoscopic per orifice approach (bronchoscope) with suction device |  | 17 |  |
| 1GM52CATJ | using per orifice approach with suction device |  | 1 |  |
| 1GZ31 | Ventilation, respiratory system NEC | Emergent | 2112 | 5 |
| 1GZ31CAMP | non-invasive ventilation bag and mask (e.g. Ambu bag) |  | 4 |  |
| 1GZ31CAND | invasive per orifice approach by endotracheal intubation and positive pressure |  | 1946 |  |
| 1GZ31CBND | non-invasive per orifice approach (e.g. by mask, nasal tubing) and positive pressure ventilation |  | 110 |  |
| 1GZ31CRND | invasive per orifice with incision approach for intubation through tracheostomy positive pressure |  | 50 |  |
| 1GZ31GPND | invasive percutaneous transluminal approach (e.g. transtracheal jet) through needle and positive pressure |  | 2 |  |
| 1HZ37 | Installation of external appliance, heart NEC | Emergent | 41 | 5 |
| 1HZ37JANN | of temporary (external) cardiac pacemaker |  | 41 |  |
| 1KE80 | Repair, abdominal arteries NEC | Elective | 30 | 5 |
| 1KE80GQNRN | using percutaneous transluminal approach and (endovascular) stent with synthetic graft [e.g. stent graft] |  | 5 |  |
| 1KE80LA | using open approach |  | 12 |  |
| 1KE80LAXXA | using open approach and autograft |  | 2 |  |
| 1KE80LAXXN | using open approach and synthetic material |  | 11 |  |
| 1VC93 | Amputation, femur | Elective | 28 | 5 |
| 1VC93LA | using simple apposition technique [e.g. suturing] (for closure of stump) |  | 21 |  |
| 1VC93LAXXA | using skin graft (for closure of stump) |  | 1 |  |
| 1VC93LAXXE | using local flap [e.g. myoplasty, osteoperiosteal flap or myodesis] (for closure of stump) |  | 6 |  |
| 1HZ09 | Stimulation, heart NEC | Emergent | 190 | 4 |
| 1HZ09GRJF | percutaneous transluminal (venous) approach using electrode with synchronized DC shock |  | 17 |  |
| 1HZ09JAFS | external approach using electrode converter/defibrillator |  | 72 |  |
| 1HZ09JAJF | external approach using electrode with synchronized DC shock |  | 81 |  |
| 1HZ09LACJ | open approach using manual massage |  | 10 |  |
| 1HZ09LAFS | open approach using electrode converter/defibrillator |  | 4 |  |
| 1HZ09LAJF | open approach using electrode with synchronized DC shock |  | 6 |  |
| 1KG57 | Extraction, arteries of leg NEC | Emergent | 138 | 4 |
| 1KG57GQFV | percutaneous transluminal approach no tissue used using atherectomy device |  | 3 |  |
| 1KG57GQFVA | percutaneous transluminal approach using autograft using atherectomy device |  | 1 |  |
| 1KG57GQGX | percutaneous transluminal approach no tissue used using device NEC |  | 7 |  |
| 1KG57GQGXN | percutaneous transluminal approach using synthetic material using device NEC |  | 3 |  |
| 1KG57LAFV | open approach using atherectomy device |  | 7 |  |
| 1KG57LAFVA | open approach using autograft using atherectomy device |  | 2 |  |
| 1KG57LAFVN | open approcah using synthetic material using atherectomy device |  | 1 |  |
| 1KG57LAGX | open approach using device NEC |  | 76 |  |
| 1KG57LAGXA | open approach using autograft using device NEC |  | 11 |  |
| 1KG57LAGXL | open approach using xenograft using device NEC |  | 4 |  |
| 1KG57LAGXN | open approach using synthetic material using device NEC |  | 23 |  |
| 1NK76 | Bypass, small intestine | Emergent | 57 | 4 |
| 1NK76DP | endoscopic [laparoscopic] approach Enteroenterostomy bypass technique |  | 1 |  |
| 1NK76RE | open approach Enterocolostomy bypass technique |  | 8 |  |
| 1NK76RF | open approach Enteroenterostomy bypass technique |  | 21 |  |
| 1NK76RJ | open approach Gastroenterostomy bypass technique |  | 26 |  |
| 1NK76SL | open approach Truncal vagotomy (or vagotomy NOS) with gastroenterostomy bypass technique |  | 1 |  |
| 1NK80 | Repair, small intestine | Emergent | 88 | 4 |
| 1NK80DA | endoscopic [laparoscopic] approach using apposition technique [e.g. suturing, stapling] |  | 4 |  |
| 1NK80DAXXE | endoscopic [laparoscopic] approach using local transposition flap [e.g. omental patch] |  | 5 |  |
| 1NK80LA | open approach using apposition technique [e.g. suturing, stapling] |  | 44 |  |
| 1NK80LAXXE | open approach using local transposition flap [e.g. omental patch] |  | 35 |  |
| 1AA52 | Drainage, meninges and dura mater of brain | Emergent | 184 | 3 |
| 1AA52HA | percutaneous approach [e.g. by needle or through fontanelle] using burr hole technique |  | 1 |  |
| 1AA52SE | open approach using burr hole technique |  | 18 |  |
| 1AA52SETS | open approach using burr hole technique and leaving drainage tube [catheter] in situ |  | 90 |  |
| 1AA52SZ | open approach using craniotomy [craniectomy] flap technique |  | 21 |  |
| 1AA52SZAZ | open approach using craniotomy [craniectomy] flap and ultrasonic device [CUSA] |  | 1 |  |
| 1AA52SZTS | open approach using craniotomy [craniectomy] flap and leaving drainage tube [catheter] in situ |  | 53 |  |
| 1AN87 | Excision partial, brain | Emergent | 136 | 3 |
| 1AN87SEAZ | burr hole technique for access with ultrasonic aspirator[e.g. CUSA] |  | 1 |  |
| 1AN87SEGX | burr hole technique for access with device NEC |  | 1 |  |
| 1AN87SZAZ | craniotomy [or craniectomy] flap technique for access with ultrasonic aspirator[e.g. CUSA] |  | 27 |  |
| 1AN87SZGX | craniotomy [or craniectomy] flap technique for access with device NEC |  | 107 |  |
| 1GY13 | Control of bleeding, thoracic cavity NEC | Emergent | 95 | 3 |
| 1GY13LA | using open approach |  | 95 |  |
| 1HA52 | Drainage, pericardium | Emergent | 60 | 3 |
| 1HA52HA | using percutaneous (needle) approach |  | 32 |  |
| 1HA52HATS | using percutaneous (needle) approach leaving drainage tube [catheter] in situ |  | 15 |  |
| 1HA52QA | using subxiphoid approach |  | 4 |  |
| 1HA52QB | using thoracic approach (e.g. sternotomy, thoracotomy) |  | 9 |  |
| 1IS51 | Occlusion, vena cava (superior and inferior) | Emergent | 60 | 3 |
| 1IS51GRKA | using filtering device percutaneous transluminal approach |  | 60 |  |
| 1NA13 | Control of bleeding, esophagus | Emergent | 66 | 3 |
| 1NA13BA | using endoscopic per orifice approach and device NEC |  | 1 |  |
| 1NA13BABD | using endoscopic per orifice approach and balloon (or Sengstaken) tube tamponade |  | 1 |  |
| 1NA13BAFA | using endoscopic per orifice approach and banding (varices) |  | 51 |  |
| 1NA13BAGX | using endoscopic per orifice approach and device NEC (e.g. electrocautery, endoclips) |  | 1 |  |
| 1NA13BAKK | using endoscopic per orifice approach and electrical heat device [e.g. argon plasma coagulation] |  | 2 |  |
| 1NA13BAX7 | using endoscopic per orifice approach and chemocautery agent |  | 8 |  |
| 1NA13DAE3 | using endoscopic [VATS] approach and cardiac stimulant (e.g. epinephrine) |  | 1 |  |
| 1NA13GQC2 | using percutaneous transluminal approach [e.g. transarterial] approach and antihemorrhagic agent |  | 1 |  |
| 1NA50 | Dilation, esophagus | Emergent | 62 | 3 |
| 1NA50BABD | using balloon dilator |  | 3 |  |
| 1NA50BABJ | using flexible dilator |  | 32 |  |
| 1NA50BABP | using rigid dilator |  | 10 |  |
| 1NA50BANR | using endoscopic per orifice approach and stent |  | 14 |  |
| 1NA50CABJ | using per orifice approach and (unguided) flexible dilator |  | 3 |  |
| 1NP13 | Control of bleeding, small and large intestine | Emergent | 85 | 3 |
| 1NP13BAC2 | using endoscopic per orifice approach and antihemorrhagic agent |  | 22 |  |
| 1NP13BAGN | using endoscopic per orifice approach and compression device [e.g. gastric balloon or bubble] |  | 3 |  |
| 1NP13BAGX | using endoscopic per orifice approach and device NEC [e.g. endoclips] |  | 12 |  |
| 1NP13BAKK | using endoscopic per orifice approach and electrical heat device [e.g. argon plasma coagulation] |  | 42 |  |
| 1NP13GQC2 | using percutaneous transluminal approach and antihemorrhagic agent |  | 3 |  |
| 1NP13GQGE | using percutaneous transluminal (transarterial) approach and [detachable] coils |  | 1 |  |
| 1NP13GQW0 | using percutaneous transluminal (transarterial) approach and synthetic agent [e.g. gelfoam, microspheres, polystyrene, polyvinyl alcohol] |  | 2 |  |
| 1VQ93 | Amputation, tibia and fibula | Emergent | 86 | 3 |
| 1VQ93LA | using simple apposition technique [e.g. suturing] (for closure of stump) |  | 42 |  |
| 1VQ93LAXXA | using skin graft (for closure of stump) |  | 1 |  |
| 1VQ93LAXXE | using local flap [e.g. myoplasty, osteoperiosteal flap or myodesis] (for closure of stump) |  | 43 |  |
| 1GJ77 | Bypass with exteriorization, trachea | Emergent | 176 | 2 |
| 1GJ77HA | using percutaneous needle approach (e.g. percutaneous dilational tracheostomy - PDT) |  | 51 |  |
| 1GJ77LA | using open approach (e.g. collar incision) |  | 62 |  |
| 1GJ77LALG | using open approach and temporary implant |  | 62 |  |
| 1GJ77QB | using mediastinal approach |  | 1 |  |
| 1GZ31 | Ventilation, respiratory system NEC | Elective | 1140 | 2 |
| 1GZ31CAND | invasive per orifice approach by endotracheal intubation and positive pressure |  | 1094 |  |
| 1GZ31CBND | non-invasive per orifice approach (e.g. by mask, nasal tubing) and positive pressure ventilation |  | 21 |  |
| 1GZ31CRND | invasive per orifice with incision approach for intubation through tracheostomy positive pressure |  | 25 |  |
| 1NF53 | Implantation of internal device, stomach | Emergent | 272 | 2 |
| 1NF53BTTS | of (gastric) tube using per orifice endoscopic approach with percutaneous incision |  | 154 |  |
| 1NF53CATS | of gastric tube [e.g. nasogastric feeding tube] using per orifice approach |  | 56 |  |
| 1NF53DATS | of (gastric) tube using endoscopic (laparoscopic) approach |  | 6 |  |
| 1NF53HATS | of (gastric) tube using percutaneous approach |  | 18 |  |
| 1NF53LATS | of (gastric) tube using open (laparotomy) approach |  | 38 |  |
| 1NK87 | Excision partial, small intestine | Emergent | 265 | 2 |
| 1NK87BA | endoscopic per orifice approach Simple excisional technique |  | 2 |  |
| 1NK87DA | endoscopic [laparoscopic] approach Simple excisional technique |  | 3 |  |
| 1NK87DN | endoscopic [laparoscopic] approach Enterocolostomy anastomosis technique |  | 5 |  |
| 1NK87DP | endoscopic [laparoscopic] approach Enteroenterostomy anastomosis technique |  | 5 |  |
| 1NK87DX | endoscopic [laparoscopic] approach Stoma formation with distal closure |  | 1 |  |
| 1NK87LA | open approach Simple excisional technique |  | 36 |  |
| 1NK87RE | open approach Enterocolostomy anastomosis technique |  | 39 |  |
| 1NK87RF | open approach Enteroenterostomy anastomosis technique |  | 150 |  |
| 1NK87TF | open approach Stoma formation with distal closure |  | 11 |  |
| 1NK87TG | open approach Stoma formation with mucous fistula |  | 13 |  |
| 1NM87 | Excision partial, large intestine | Emergent | 404 | 2 |
| 1NM87BA | endoscopic per orifice approach Simple excisional technique |  | 68 |  |
| 1NM87DA | endoscopic [laparoscopic] approach Simple excisional technique |  | 7 |  |
| 1NM87DE | endoscopic [laparoscopic] approach Colorectal anastomosis technique |  | 4 |  |
| 1NM87DF | endoscopic [laparoscopic] approach Colocolostomy anastomosis technique |  | 5 |  |
| 1NM87DN | endoscopic [laparoscopic] approach Enterocolostomy anastomosis technique |  | 11 |  |
| 1NM87DX | endoscopic [laparoscopic] approach Stoma formation and distal closure |  | 2 |  |
| 1NM87DY | endoscopic [laparoscopic] approach Stoma formation with creation of mucous fistula |  | 1 |  |
| 1NM87LA | open approach Simple excisional technique |  | 26 |  |
| 1NM87RD | open approach Colorectal anastomosis technique |  | 18 |  |
| 1NM87RE | open approach Enterocolostomy anastomosis technique |  | 116 |  |
| 1NM87RN | open approach Colocolostomy anastomosis technique |  | 57 |  |
| 1NM87TF | open approach Stoma formation with distal closure |  | 75 |  |
| 1NM87TG | open approach Stoma formation with creation of mucous fistula |  | 14 |  |
| 1OT52 | Drainage, abdominal cavity | Emergent | 579 | 2 |
| 1OT52CQ | using per orifice [transvaginal] needle aspiration technique |  | 1 |  |
| 1OT52DA | using endoscopic (laparoscopic) approach |  | 9 |  |
| 1OT52DATS | using endoscopic (laparoscopic) approach and leaving drainage tube in situ |  | 14 |  |
| 1OT52HA | using percutaneous (needle) approach |  | 214 |  |
| 1OT52HATS | using percutaneous (needle) approach and and leaving drainage tube in situ |  | 185 |  |
| 1OT52HHD1 | using percutaneous transcatheter approach and anti infective irrigating solution |  | 4 |  |
| 1OT52HHD2 | using percutaneous transcatheter approach and salt irrigating solution |  | 1 |  |
| 1OT52HHD3 | using percutaneous transcatheter approach and other irrigating solution |  | 17 |  |
| 1OT52LA | using open approach |  | 80 |  |
| 1OT52LATS | using open (incisional) approach and leaving drainage tube in situ |  | 54 |  |
| 1VA53 | Implantation of internal device, hip joint | Emergent | 570 | 2 |
| 1VA53LAPM | open approach uncemented single-component prosthetic device [femoral] |  | 330 |  |
| 1VA53LAPMA | open approach using bone autograft [uncemented] single-component prosthetic device [femoral] |  | 40 |  |
| 1VA53LAPMK | open approach using bone homograft [uncemented] single-component prosthetic device [femoral] |  | 2 |  |
| 1VA53LAPMN | open approach cemented single-component prosthetic device [femoral] |  | 74 |  |
| 1VA53LAPMQ | open approach using combined bone graft and cement or paste single-component prosthetic device [femoral] |  | 2 |  |
| 1VA53LAPN | open approach uncemented dual-component prosthetic device [femoral & acetabular] |  | 64 |  |
| 1VA53LAPNA | open approach using bone autograft [uncemented] dual-component prosthetic device [femoral & acetabular] |  | 18 |  |
| 1VA53LAPNK | open approach using bone homograft [uncemented] dual-component prosthetic device [femoral & acetabular] |  | 2 |  |
| 1VA53LAPNN | open approach cemented dual-component prosthetic device [femoral & acetabular] |  | 27 |  |
| 1VA53LAPNQ | using combined bone graft and cement or paste dual-component prosthetic device [femoral & acetabular] |  | 8 |  |
| 1VA53LASLN | open approach cement spacer [temporary, impregnated with antibiotics] |  | 3 |  |
| 1VC74 | Fixation, femur | Emergent | 694 | 2 |
| 1VC74HALQ | percutaneous approach [e.g. with closed reduction or no reduction] fixation device alone using intramedullary nail |  | 20 |  |
| 1VC74HANV | percutaneous approach [e.g. with closed reduction or no reduction] fixation device alone using pin, nail |  | 5 |  |
| 1VC74HANW | using percutaneous approach [e.g with closed reduction or no reduction] using plate/screw |  | 14 |  |
| 1VC74LAKD | open approach fixation device alone using wire, staple |  | 26 |  |
| 1VC74LAKDA | open approach with bone autograft using wire, staple |  | 2 |  |
| 1VC74LAKDK | open approach with bone homograft using wire, staple |  | 4 |  |
| 1VC74LAKDN | open approach with synthetic tissue [e.g. bone cement, or paste] using wire, staple |  | 1 |  |
| 1VC74LALQ | open approach fixation device alone using intramedullary nail |  | 224 |  |
| 1VC74LALQA | open approach with bone autograft using intramedullary nail |  | 5 |  |
| 1VC74LALQK | open approach with bone homograft using intramedullary nail |  | 2 |  |
| 1VC74LALQN | open approach with synthetic tissue [e.g. bone cement, or paste] using intramedullary nail |  | 8 |  |
| 1VC74LALQQ | open approach with combined sources of tissue [e.g. graft & cement/paste] using intramedullary nail |  | 1 |  |
| 1VC74LANV | open approach fixation device alone using pin, nail |  | 33 |  |
| 1VC74LANVQ | open approach with combined sources of tissue [e.g. graft & cement/paste] using pin, nail |  | 1 |  |
| 1VC74LANW | open approach fixation device alone using screw, plate and screw |  | 327 |  |
| 1VC74LANWA | open approach with bone autograft using screw, plate and screw |  | 4 |  |
| 1VC74LANWK | open approach with bone homograft using screw, plate and screw |  | 7 |  |
| 1VC74LANWN | open approach with synthetic tissue [e.g. bone cement, or paste] using screw, plate and screw |  | 7 |  |
| 1VC74LANWQ | open approach with combined sources of tissue [e.g. graft & cement/paste] using screw, plate and screw |  | 3 |  |
| 1VC93 | Amputation, femur | Emergent | 86 | 2 |
| 1VC93LA | using simple apposition technique [e.g. suturing] (for closure of stump) |  | 68 |  |
| 1VC93LAXXE | using local flap [e.g. myoplasty, osteoperiosteal flap or myodesis] (for closure of stump) |  | 18 |  |
| 1GV52 | Drainage, pleura | Emergent | 849 | 1 |
| 1GV52DA | using endoscopic approach [VATS] |  | 8 |  |
| 1GV52DATS | using endoscopic approach and leaving drainage tube in situ |  | 21 |  |
| 1GV52HA | using percutaneous (needle) approach |  | 149 |  |
| 1GV52HAHE | using percutaneous catheter (intracostal) with underwater seal drainage system |  | 290 |  |
| 1GV52HATK | using percutaneous catheter with suction pump, (under water seal or negative pressure) |  | 323 |  |
| 1GV52LA | using open approach |  | 20 |  |
| 1GV52LATS | using open approach and leaving drainage tube in situ |  | 38 |  |
| 1IS53 | Implantation of internal device, vena cava (superior and inferior) | Emergent | 1531 | 1 |
| 1IS53GRLF | vascular access device with external lumen using percutaneous transluminal venous approach |  | 1226 |  |
| 1IS53HNLF | vascular access device using percutaneous tunnelling technique |  | 240 |  |
| 1IS53LALF | totally implanted venous access device (with injection port)[e.g Port-a-cath] using open approach |  | 65 |  |
| 1IL35 | Pharmacotherapy (local), vessels of heart | Emergent | 1629 | -2 |
| 1IL35HAC1 | percutaneous injection approach of antithrombotic agent |  | 22 |  |
| 1IL35HAT9 | percutaneous injection approach of pharmacological agent NEC |  | 8 |  |
| 1IL35HHC1 | percutaneous infusion approach of antithromobtic agent |  | 754 |  |
| 1IL35HHM8 | percutaneous infusion approach of immunosuppressive agent |  | 286 |  |
| 1IL35HHT9 | percutaneous infusion approach of pharmacological agent NEC |  | 559 |  |
| 1NV89 | Excision total, appendix | Emergent | 948 | -3 |
| 1NV89DA | using endoscopic [laparoscopic] approach |  | 644 |  |
| 1NV89LA | using open approach |  | 304 |  |
| 1IJ50 | Dilation, coronary arteries | Elective | 978 | -4 |
| 1IJ50GQBD | using percutaneous transluminal approach and balloon dilator |  | 61 |  |
| 1IJ50GQBF | using percutaneous transluminal approach with laser and balloon dilator |  | 1 |  |
| 1IJ50GQNR | using percutaneous transluminal approach and (endovascular) stent (insertion) |  | 6 |  |
| 1IJ50GQOA | using percutaneous transluminal approach and balloon dilator with (endovascular) stent (insertion) |  | 904 |  |
| 1IJ50GQOE | using percutaneous transluminal approach, therapeutic ultrasound, balloon dilator and (endovascular) stent (insertion) |  | 6 |  |
| 1LZ37 | Installation of external appliance, circulatory system NEC | Emergent | 952 | -4 |
| 1LZ37GPGB | percutaneous transluminal (closed chest) approach cardiopulmonary bypass (intraoperative) |  | 1 |  |
| 1LZ37GPQM | percutaneous transluminal (closed chest) approach extracorporeal membrane oxygenator [ECMO] |  | 1 |  |
| 1LZ37LAFP | extracorporeal blood salvage device |  | 37 |  |
| 1LZ37LAGB | extracorporeal circulation device |  | 912 |  |
| 1LZ37LAQM | extracorporeal membrane oxygenator [ECMO] |  | 1 |  |
| 1VA53 | Implantation of internal device, hip joint | Elective | 1384 | -4 |
| 1VA53LAPM | open approach uncemented single-component prosthetic device [femoral] |  | 9 |  |
| 1VA53LAPMA | open approach using bone autograft [uncemented] single-component prosthetic device [femoral] |  | 5 |  |
| 1VA53LAPMK | open approach using bone homograft [uncemented] single-component prosthetic device [femoral] |  | 3 |  |
| 1VA53LAPMN | open approach cemented single-component prosthetic device [femoral] |  | 11 |  |
| 1VA53LAPMQ | open approach using combined bone graft and cement or paste single-component prosthetic device [femoral] |  | 3 |  |
| 1VA53LAPN | open approach uncemented dual-component prosthetic device [femoral & acetabular] |  | 666 |  |
| 1VA53LAPNA | open approach using bone autograft [uncemented] dual-component prosthetic device [femoral & acetabular] |  | 346 |  |
| 1VA53LAPNK | open approach using bone homograft [uncemented] dual-component prosthetic device [femoral & acetabular] |  | 20 |  |
| 1VA53LAPNN | open approach cemented dual-component prosthetic device [femoral & acetabular] |  | 211 |  |
| 1VA53LAPNQ | using combined bone graft and cement or paste dual-component prosthetic device [femoral & acetabular] |  | 107 |  |
| 1VA53LASLN | open approach cement spacer [temporary, impregnated with antibiotics] |  | 3 |  |
| 1VG53 | Implantation of internal device, knee joint | Elective | 1816 | -4 |
| 1VG53LAPM | uncemented single component prosthetic device [unicondylar] |  | 17 |  |
| 1VG53LAPMA | with bone autograft single component prosthetic device [unicondylar] |  | 2 |  |
| 1VG53LAPMN | cemented single component prosthetic device [unicondylar] |  | 158 |  |
| 1VG53LAPMQ | with combined bone graft and cement or paste single component prosthetic device [unicondylar] |  | 10 |  |
| 1VG53LAPN | uncemented dual component prosthetic device [bicondylar] |  | 11 |  |
| 1VG53LAPNA | with bone autograft dual component prosthetic device [bicondylar] |  | 3 |  |
| 1VG53LAPNN | cemented dual component prosthetic device [bicondylar] |  | 226 |  |
| 1VG53LAPNQ | with combined bone graft and cement or paste dual component prosthetic device [bicondylar] |  | 30 |  |
| 1VG53LAPP | uncemented tri component prosthetic device [medial, lateral & patellofemoral] |  | 46 |  |
| 1VG53LAPPA | with bone autograft tri component prosthetic device [medial, lateral & patellofemoral] |  | 23 |  |
| 1VG53LAPPN | cemented tri component prosthetic device [medial, lateral & patellofemoral] |  | 842 |  |
| 1VG53LAPPQ | with combined bone graft and cement or paste tri component prosthetic device [medial, lateral & patellofemoral] |  | 438 |  |
| 1VG53LASLN | cemented cement spacer (temporary) [impregnated with antibiotics] |  | 10 |  |
| 1RD89 | Excision total, ovary with fallopian tube | Elective | 1774 | -5 |
| 1RD89DA | using endoscopic [laparoscopic] approach |  | 341 |  |
| 1RD89LA | using open approach |  | 1393 |  |
| 1RD89RA | using open vaginal approach |  | 40 |  |
| 1SY80 | Repair, muscles of the chest and abdomen | Elective | 2846 | -5 |
| 1SY80DA | endoscopic [laparoscopic] approach without tissue [e.g. suturing or stapling] |  | 75 |  |
| 1SY80DAXXN | endoscopic [laparoscopic] approach using synthetic tissue [e.g. mesh, sponge] |  | 100 |  |
| 1SY80LA | open approach without tissue [e.g. suturing or stapling] |  | 387 |  |
| 1SY80LATZ | open approach using zipper [temporary] (for repeat access to abdomen) |  | 1 |  |
| 1SY80LAXXA | open approach using autograft [e.g. fascia, skin] |  | 11 |  |
| 1SY80LAXXG | open approach using pedicled flap [e.g. abdominis rectus or deltopectoral] |  | 1 |  |
| 1SY80LAXXN | open approach using synthetic tissue [e.g. mesh, sponge] |  | 2256 |  |
| 1SY80LAXXQ | open approach and combined sources of tissue (e.g. mesh with autograft) |  | 9 |  |
| 1SY80WJ | open approach using special excisional technique |  | 6 |  |
| 1OT87 | Excision partial, abdominal cavity | Emergent | 62 | -6 |
| 1OT87DA | using endoscopic [laparoscopic] approach |  | 2 |  |
| 1OT87LA | using open approach |  | 60 |  |
| 1QT87 | Excision partial, prostate | Elective | 773 | -6 |
| 1QT87BA | endoscopic per orifice approach (TURP) using device NEC |  | 758 |  |
| 1QT87BAAG | endoscopic per orifice [transurethral] approach Using laser NEC |  | 1 |  |
| 1QT87BAAK | endoscopic per orifice approach (transurethral) Using loop electrode |  | 8 |  |
| 1QT87PB | open perineal approach Using device NEC (e.g. digital dissection) |  | 2 |  |
| 1QT87PK | open retropubic approach Using device NEC (e.g. digital dissection) |  | 3 |  |
| 1QT87QZ | open transvesical approach Using device NEC (e.g. digital dissection) |  | 1 |  |
| 1RM89 | Excision total, uterus and surrounding structures | Elective | 2184 | -7 |
| 1RM89AA | using combined laparoscopic and vaginal approach |  | 81 |  |
| 1RM89CA | using vaginal approach |  | 639 |  |
| 1RM89DA | using endoscopic (laparoscopic) approach |  | 47 |  |
| 1RM89LA | using open approach |  | 1417 |  |
